# Water availability drives gas exchange and growth of trees in northeastern US, not elevated CO_2_ and reduced acid deposition

**DOI:** 10.1038/srep46158

**Published:** 2017-04-10

**Authors:** Mathieu Levesque, Laia Andreu-Hayles, Neil Pederson

**Affiliations:** 1 Tree-Ring Laboratory, Lamont-Doherty Earth Observatory of Columbia University, Palisades, NY 10964, USA; 2 Harvard Forest, Harvard University, Petersham, MA 01366, USA

## Abstract

Dynamic global vegetation models (DGVM) exhibit high uncertainty about how climate change, elevated atmospheric CO_2_ (atm. CO_2_) concentration, and atmospheric pollutants will impact carbon sequestration in forested ecosystems. Although the individual roles of these environmental factors on tree growth are understood, analyses examining their simultaneous effects are lacking. We used tree-ring isotopic data and structural equation modeling to examine the concurrent and interacting effects of water availability, atm. CO_2_ concentration, and SO_4_ and nitrogen deposition on two broadleaf tree species in a temperate mesic forest in the northeastern US. Water availability was the strongest driver of gas exchange and tree growth. Wetter conditions since the 1980s have enhanced stomatal conductance, photosynthetic assimilation rates and, to a lesser extent, tree radial growth. Increased water availability seemingly overrides responses to reduced acid deposition, CO_2_ fertilization, and nitrogen deposition. Our results indicate that water availability as a driver of ecosystem productivity in mesic temperate forests is not adequately represented in DGVMs, while CO_2_ fertilization is likely overrepresented. This study emphasizes the importance to simultaneously consider interacting climatic and biogeochemical drivers when assessing forest responses to global environmental changes.

Long-term changes in tree growth and forest productivity have been attributed to multiple climatic and biogeochemical drivers including regional changes in temperature and precipitation regimes, elevated atm. CO_2_ concentration, nitrogen deposition, and atmospheric pollution^1^,^2^. Still, a critical question remains “*What are the simultaneous impacts of divergent and interacting environmental drivers of forest productivity*?” Although moderate warming, higher atm. CO_2_ concentration, and nitrogen deposition can enhance forest productivity and carbon sequestration^3^,^4^, increased heat^5^, drought^6^, and atmospheric pollution^2^ could counteract these positive effects. Disentangling the impact of these drivers on forest productivity is crucial for better anticipating future changes in biogeochemical cycles and ecosystem services.

One region where the simultaneous influence of multiple environmental drivers on forest productivity can be tested is the northeastern United States (US). Over the last decades, this region has experienced simultaneous and significant shifts in moisture availability^7^, increases in atm. CO_2_ concentration, and reductions in acid and nitrogen deposition^4^,^8^ (Fig. 1). While there has been a substantial reduction in acid deposition in northeastern US, there is still not a consensus that reduced pollutant loads have enhanced tree growth in temperate mesic forests^9^,^10^,^11^. The simultaneous increase in water availability and decrease in acid deposition complicate our understanding of the potential benefits of reduced acid deposition. At the same time, the divergent influences between moisture stress and potential CO_2_ fertilization have led to significant disagreement between remotely sensed (satellite) and modeled (DGVM) productivity of temperate mesic forests^12^. Investigating the concurrent effects of varying environmental drivers on growth and gas exchange of trees is critical to improve DGVM and better understand the rates, magnitude, and trajectory of terrestrial carbon budgets.

Trees acclimate to environmental changes at the leaf level by adjusting their stomatal conductance (*g*_*s*_) and photosynthetic assimilation rates (*A*). These adjustments translate into changes in allocation and growth^13^,^14^. Concurrent adjustments at the tree level interact and influence transpiration and carbon assimilation rates from stand to landscape scales^15^. Long-term information on physiological and environmental processes at annual and seasonal time-scales can be gained through stable isotopic analysis of tree rings^16^,^17^. Stable isotopic analysis can be used to assess how stomatal conductance and photosynthesis respond to shifts in moisture availability^18^, increasing CO_2_ concentration^19^, and reductions in acid deposition^20^.

Here, we assess the simultaneous effects of changes in key environmental factors on gas exchange and tree growth in a temperate mesic forest of northeastern US using isotopic records from tree rings of two dominant and widely distributed tree species in eastern North America, *Liriodendron tulipifera* L. and *Quercus rubra* L. (see Methods, Fig. 1, Supplementary Table S1). Under dry conditions, *L. tulipifera* has an isohydric behavior and constrains its stomatal conductance so that mid-day water potential minima is kept below a critical threshold^21^. In contrast, *Q. rubra* shows an anisohydric behavior and maintains constant levels of stomatal conductance during drought at the risk of incurring xylem cavitation^21^. Contrasting physiological behavior and habitats of our study trees make them ideal for isolating growth and physiological responses to concurrent but divergent changes in key environmental factors. We first assess the simultaneous influences of changes in atmospheric CO_2_ concentration, climatic water balance, and SO_4_ and N deposition on tree growth and physiological mechanisms with structural equation models (SEM, Fig. 2). Second, we analyze the growth, carbon isotope discrimination (Δ^13^C), intrinsic water-use efficiency (_i_WUE), and oxygen isotopic ratio (δ^18^O) responses of trees to shifting moisture conditions, from the extreme 1960s drought to repeated pluvial periods since the 1980s (Fig. 1c).

## Results and Discussion

Hypothetically, broadleaf trees in temperate mesic forests are sensitive to moisture availability because of poor stomatal regulation, low hydraulic conductance, high leaf area, and the high radiation and evaporative demands experienced by their large crowns^22^. We found support for this hypothesis through the high sensitivity of mature (>100 yrs old) *L. tulipifera* and *Q. rubra* to moisture availability irrespective of their physiological behavior (isohydric vs anisohydric) and site conditions (moist lowland vs shallow-soiled ridge site). Particularly, we observed a strong coupling between moisture availability and gas exchange of trees as indicated by the strong correlations between the isotopic tree-ring data and summer climatic water balance (Δ^13^C, *r* = 0.73 and 0.65; δ^18^O, *r* = −0.71 and −0.55) and maximum vapor pressure deficit (Δ^13^C, *r* = −0.70 and −0.70; δ^18^O, *r* = 0.71 and 0.56) (Table 1). Such high correlations indicate an even greater sensitivity of temperate broadleaf trees to drought than a recent analysis over the eastern US^23^. Given that our period of study covers one of the wettest periods of the last 500 years^7^, if not the last 3000–5000 years^24^, the strong sensitivity of tree gas exchange and to a lower degree of growth to moisture availability in this mesic region is particularly striking.

Elevated atm. CO_2_ concentration has been found to stimulate tree growth by indirectly enhancing photosynthetic rates and _i_WUE^25^,^26^. When simultaneously analyzing tree sensitivity to summer climatic water balance, atm. CO_2_ concentration, and SO_4_ and N deposition, however, we found that water availability was the most important factor. Climatic water balance during the summer (June, July, August) was the strongest driver of BAI, Δ^13^C, _i_WUE, and δ^18^O in both species (Fig. 3). In contrast, atm. CO_2_ concentration, SO_4_, and N deposition, which showed significant covariation, exhibited negligible effects. Even if elevated atm. CO_2_ concentration directly improved _i_WUE (although this is partly due to the inclusion of CO_2_ in _i_WUE calculation, Supplementary Methods S1, eqn. 3), the inexistent or negative associations found between _i_WUE and BAI, as well as between atm. CO_2_ concentration and BAI indicate little to no stimulation of growth to CO_2_. These findings match experiments in mature forests where elevated atm. CO_2_ concentration and increases in _i_WUE do not necessarily translate into enhanced radial tree growth^27^. In those settings, heat and drought stress^16^,^28^,^29^ or limited nutrient availability^30^ override CO_2_ effect. The lack of evidence of CO_2_ fertilization effect on tree growth in our study cannot be attributed to moisture deficit or warming-induced drought stress because the period of the SEM analysis (1981–2014) is the wettest period of the instrumental period that began in 1895. Therefore, our results indicate that CO_2_-induced growth enhancement^25^ is unlikely for mature trees under natural conditions when the effects of the concomitant and significant covariation in climatic water balance, SO_4_ deposition, and N deposition are considered.

Acid deposition can alter leaf physiology and stomatal conductance, indirectly modify isotope ratios in tree rings^20^, and influence tree growth^9^. When we simultaneously analyzed the effects of the climatic water balance, atm. CO_2_ concentration, and atmospheric deposition on trees, we did not detect any direct effect of SO_4_ and N deposition on BAI and only found some direct but small effects of SO_4_ and N deposition on Δ^13^C, _i_WUE, and δ^18^O (Fig. 3). However, these direct effects on tree-ring isotopic ratios were not translated into changes in growth. Inexistent or negative correlations were found between Δ^13^C or _i_WUE and BAI. *L. tulipifera*, a species with arbuscular mycorrhizal association, may show higher growth to increased availability of inorganic N from atmospheric deposition^4^,^31^. However, the SEM indicated no direct positive association between N deposition and BAI of *L. tulipifera*. Similarly, the BAI of *Q. rubra* did not show any direct association with SO_4_ and N deposition despite the presence of some minor correlations between tree-ring Δ^13^C and δ^18^O with N and SO_4_, respectively. These results do not support previous findings that showed a high sensitivity of this species to S and N deposition^32^ and N-induced growth enhancement under natural conditions^4^,^33^. The absence of growth response to N deposition found for both species agrees with the results of an N-addition experiment done in the Catskill Mountains of southeastern New York State where N addition had no significant effects on aboveground biomass production^34^.

Our SEM analysis indicates that moisture is the primary driver of gas exchange and growth for both species, even during an anomalously wet period. From these analyses, the increase in forest growth recently observed in the northeastern US^4^,^9^,^35^ is likely less related to rising atm. CO_2_ concentration and changes in acid/nitrogen deposition and more likely driven by regional wetting^7^,^35^. Supporting this inference, change-point detection analysis of the climatic water balance time-series identified a tipping point in moisture availability with drier conditions prior to 1983 and wetter after (Fig. 4a,b). Concurrent to this water availability increase, *L. tulipifera* and *Q. rubra* exhibited a simultaneous shift in BAI, Δ^13^C, _i_WUE, and δ^18^O (Fig. 4) indicating a strong coupling between growth, gas exchange, and moisture conditions. A similar increase in tree-ring Δ^13^C and enhancement in growth was found in the *Q. rubra* Harvard Forest eddy-flux tower forest following the regional increased in water availability^35^.

While BAI, Δ^13^C, and _i_WUE mainly increased as the climate became wetter, tree-ring δ^18^O started to decrease (Fig. 4) because of changes in isotopic composition of water sources and stomatal response to lower evaporative demand of the atmosphere. Variation in tree-ring δ^18^O is primarily due to evaporative enrichment at the leaf level, biochemical fractionation during oxygen incorporation, and isotopic signature of tree source water, which is mainly influenced by the δ^18^O of precipitation and soil evaporative enrichment^36^,^37^. The δ^18^O value of precipitation is essentially influenced by air temperature, precipitation amount, moisture sources, air mass trajectory, and seasonality^38^.

In our study region, the δ^18^O of precipitation has changed through time (Supplementary Methods S2, Fig. S1). Between 1968 and 2010, a significant reduction in δ^18^O of precipitation (−0.089‰ yr^−1^) was recorded in northeastern US due in large part to the increase in the proportion of Arctic precipitation sources which are more depleted in δ^18^O ratios^38^. This decrease in δ^18^O of precipitation may have potentially influenced the isotopic signature of the source water of our trees and caused a gradual reduction in tree-ring δ^18^O values through time (Fig. 4i). However, the strong coupling between tree-ring δ^18^O and moisture availability (Table 1) indicates that the decrease in tree-ring δ^18^O was also due to a reduction in transpiration at the leaf level in response to the lower evaporative demand of the atmosphere as the climate became wetter.

Synchronically to the decrease in δ^18^O tree-ring values, rising Δ^13^C and BAI (Fig. 4e,c) suggest that C-assimilation and stomatal conductance also increased. Taken together, the concurrent depletion of δ^18^O and increase in Δ^13^C indicate that, despite the reduction in δ^18^O of precipitation over the study region, changes in transpiration, stomatal conductance, and photosynthetic assimilation rates occurred simultaneously and tracked the abrupt shift in climatic water balance that began in the early 1980s. The long-term trends recorded in our tree-ring δ^18^O time series, however, should be interpret with caution as changes in the isotopic signature of source water in time and the reduction in evaporative enrichment at the leaf level driven by the increase in water availability have likely occurred at the same time.

Overall, we found that moisture is the main driver of increased gas exchange and, to a lesser degree, increased radial growth of two broadleaf trees in a mesic temperate forest in the northeastern US during the period of opposing trajectories of acid and N deposition, atm. CO_2_ concentration, and water availability. Simultaneous analysis of these drivers on tree-ring isotopic composition and growth indicates that the reported growth recovery from reduced acid deposition^9^ and N-induced growth enhancement in northeastern US forests^33^ might be the result of a possible omission of the concurrent shift in water availability and atm. CO_2_ concentration. Additionally, results here do not find support for atm. CO_2_ fertilization on broadleaf tree growth in mature temperate mesic forests of northeastern US^25^, a region where greening trends have been mainly attributed to increased atm. CO_2_ concentration and land cover change^1^. Our work emphasizes the need to simultaneously consider changes in water availability, atm. CO_2_ concentration, and acid/nitrogen deposition at large spatial scales to gain a more complete understanding of future changes in forest productivity.

As the climate is getting warmer and wetter in northeastern US (Supplementary Fig. S2), our observed sensitivity of broadleaf trees to moisture availability is important. The expected increases in severity, frequency and duration of drought periods would likely have a significant impact on tree growth, mortality rates, and forest composition^39^. Although the temperate mesic forests of the northeastern US have not experienced severe and long-lasting drought since the 1960s^7^, the strong and persistent response of trees to water availability reported here as well as in recent studies^35^,^39^ reveals a vulnerability of mesic forests to drought.

## Methods

### Study sites and dendrochronological analysis

We conducted this study at Black Rock Forest (41°24′N, 74°01′W), a 1550 ha forest preserve in southeastern New York State^40^. We sampled *L. tulipifera* in a lowland site located on a south-facing slope at 170 m a.s.l. on loamy soils and *Q. rubra* at the ridge of an upper slope site at 400 m a.s.l., situated 2 km away from the lowland site, and characterized by shallow soils with abundant rock outcrops. We extracted two 5 mm diameter increment cores from 15 dominant and healthy *L. tulipifera* and *Q. rubra* trees for tree-ring width measurements (Supplementary Table S1). The increment cores were air dried, glued on wood mounts, and successively sanded with finer grades of sandpaper until the xylem structure and ring boundaries were clearly visible. We measured ring widths to the nearest 0.001 mm. Individual tree-ring width series were crossdated and statistically checked with the program COFECHA^41^. To ensure that the number of trees sampled was sufficient and representative of the sampled population, we calculated the express population signal (EPS). All tree-ring chronologies showed EPS values ≥ 0.85, which is considered the threshold value for adequately reflecting a common signal among trees (Supplementary Table S1)^42^. To detect long-term changes in growth, we converted the individual raw tree-ring width series to basal area increments (BAI) and removed the potential age related trends that can bias long-term growth changes with a Regional Curve Standardization (RCS) approach (Supplementary Fig. S3)^43^. We calculated an average ontogenetic growth curve for each species (i.e., the regional curve) by aligning the raw BAI measurements of each tree to the biological age of the rings. We then divided each raw individual BAI series by this average curve to produce RCS residual BAI series^44^,^45^. RCS residual BAI series were used in further analyses.

### Isotopic analysis

Tree-ring δ^13^C was used to calculate carbon isotope discrimination (Δ^13^C, Supplementary Methods S1). Δ^13^C in tree rings provides an integrated record between intercellular and atm. CO_2_ concentration during the period when the carbon was fixed by the enzyme Ribulose-1,5-bisphosphate carboxylase/oxygenase (RuBisCO) in chloroplasts^46^. With further calculations, _i_WUE, i.e., the ratio between *A* and *g*_*s*_, can be determined from Δ^13^C (Supplementary Methods S1)^47^. By contrast, the oxygen isotopic ratios in tree rings integrate the isotopic composition of source water and the stomatal response to changes in vapor pressure deficit^36^,^37^. Thus, the δ^18^O ratio contains an indirect record of *g*_*s*_ and can help understanding the influence of *A* and *g*_*s*_ on Δ^13^C and _i_WUE^18^. Therefore, by examining concurrent variations in both Δ^13^C and δ^18^O, insights can be gained on how stomatal conductance and photosynthesis respond to shifts in climatic water balance, increasing atm. CO_2_ concentration, and reductions in acid deposition.

For the isotopic analysis, we selected the five trees per species with the highest correlations with the tree-ring width master chronology and took an extra 12 mm diameter core per tree. We analyzed the isotopic ratios in each tree and each annual ring individually for the period 1950–2014. From each core, we split off the latewood of each annual ring with a scalpel under a stereomicroscope, chopped the material, and stored each latewood sample individually in centrifugal tubes before cellulose extraction. We extracted the α-cellulose following standard procedures^48^,^49^ and homogenized the cellulose using an ultrasound treatment^50^. For each sample, 200 μg of cellulose were weighted and put in silver capsules. δ^13^C and δ^18^O were measured simultaneously using high-temperature pyrolysis in a Costech elemental analyzer interfaced with an Elementar Isoprime mass spectrometer at the Department of Geology at the University of Maryland, USA^51^. The analytical precision for the in-house α-cellulose standards was ±0.17‰ for δ^13^C and ±0.34‰ for δ^18^O.

### Climate and atmospheric deposition data

Modeled mean monthly minimum and maximum temperature, total monthly precipitation and maximum vapor pressure deficit for the period 1895–2014 were obtained from the PRISM Climate Group, Oregon State University (http://prism.oregonstate.edu). We used the mean monthly minimum and maximum temperature and total precipitation to compute a monthly climatic water balance, i.e., precipitation minus potential evapotranspiration. Evapotranspiration was calculated according to Hargreaves^52^.

**Figure 1:**
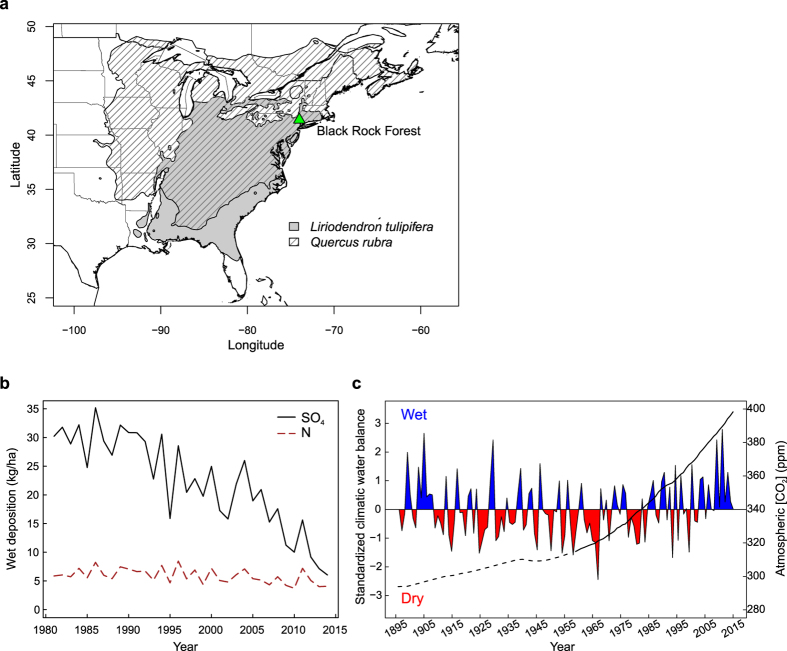
Location of the study site and time series of the SO_4_ and total inorganic N deposition, climatic water balance, and atmospheric CO_2_ concentration. **(a)** Location of Black Rock Forest and distribution range of *Liriodendron tulipifera* L. and *Quercus rubra* L. in North America. Data source: https://gec.cr.usgs.gov/data/little/. **(b)** Total SO_4_ and inorganic N wet deposition per hectare for the water year (previous October to current September) at Black Rock Forest, NY. Deposition data were obtained from the National Acid Deposition Program (http://nadp.sws.uiuc.edu); 1981 to 1983 data are from Station NY51, located 6 km from Black Rock Forest, while 1984–2014 data are from Station NY99, located at Black Rock Forest. **(c)** Standardized climatic water balance during the summer (June–August) calculated as the difference between precipitation and potential evapotranspiration. Positive values in blue indicate wet conditions and negative values in red show dry conditions. Estimated atmospheric CO_2_ concentrations from ice core data for the period 1895–1958 (dashed black line) and measured data from 1958 to 2014 (black line), CO_2_ data source: http://www.columbia.edu/~mhs119/GHGs/CO2.1850-2015.txt. Figure created with R version 3.2.2^61^.

To assess the potential effect of atm. CO_2_ concentration and pollutants on tree growth and physiology, we used historical SO_4_ and total inorganic N wet deposition data (kg/ha) for the water year (previous October to current September) from two measuring stations from the National Acid Deposition Program (Fig. 1, http://nadp.sws.uiuc.edu), and annual global average CO_2_ mixing ratio values available online (http://www.columbia.edu/~mhs119/GHGs/CO2.1850-2015.txt).

### Data analysis

We first explored the relationships of tree-ring chronologies (BAI, Δ^13^C, δ^18^O) with monthly climate variables with bootstrapped correlation functions using the R package *treeclim*^53^. We identified the months (i.e., June, July, and August) that have the strongest and significant influence on tree-ring chronologies (Supplementary Figs S4, S5) and further averaged the climate variables over these months and calculated correlation coefficients between summer climate and tree-ring variables (Table 1). Tree-ring and climate time series were prewhitened (removal of the first order autocorrelations) before correlation analysis. The summer climatic water balance was used in further analyses since it showed the strongest correlations with the tree-ring variables.

**Figure 2:**
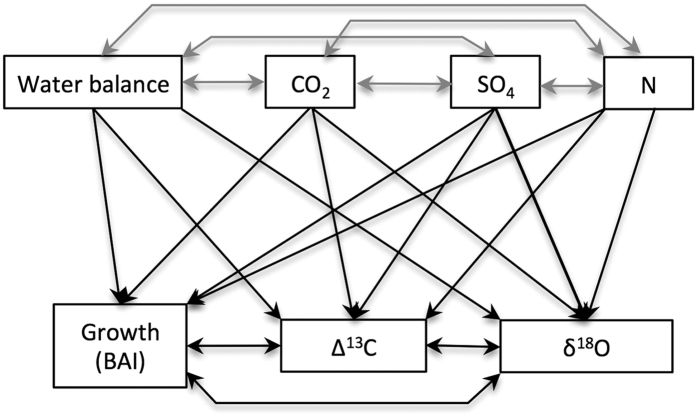
Hypothetical structural equation model used for assessing the influence of the summer climatic water balance, atmospheric CO_2_ concentration, SO_4_ and N wet deposition on tree growth (basal area increment, BAI), and tree gas exchange inferred from Δ^13^C and δ^18^O measured in tree rings. Single-headed arrows indicate causal relationships and double-headed arrows denote covariation between response variables. Grey paths indicate covariation between explanatory variables.

**Figure 3:**
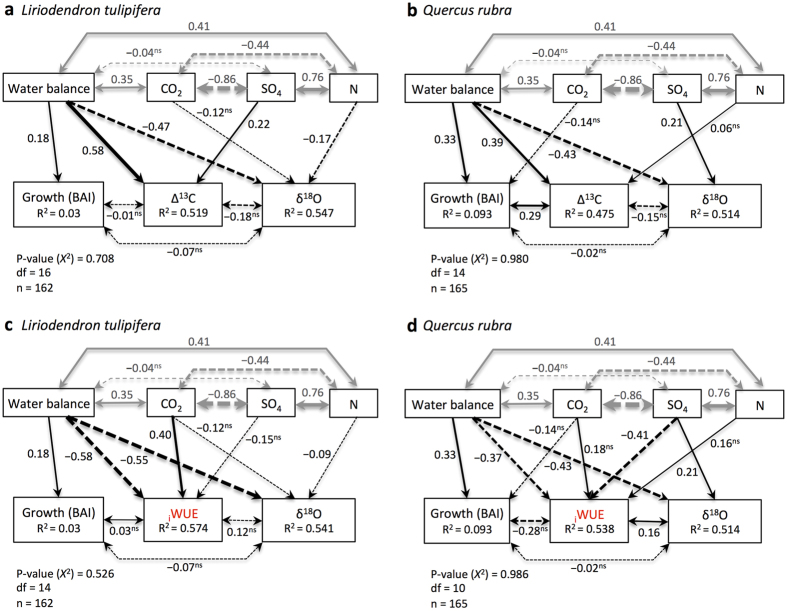
Fitted piecewise structural equation models showing the relative influence of the summer climatic water balance, atmospheric CO_2_ concentration, and SO_4_ and N wet deposition on tree growth inferred from basal area increment (BAI), Δ^13^C (**a,b**), _i_WUE (**c,d**), and δ^18^O. Period of analysis 1981–2014. The random tree identity effect (5 trees per species) was considered by fitting each response variable to a linear mixed effects model within the structural equation models. Single-headed arrows indicate causal relationships and double-headed arrows denote covariation between variables. The width of arrows is proportional to the strength of path coefficients. Numbers next to the paths indicate standardized path coefficients. Coefficients with ^ns^ are not significant, but improved the model fit. Solid and dashed paths indicate positive and negative effects, respectively. Grey paths indicate covariation between explanatory variables. Amount of variance explained by the model (R^2^) is listed for each response variable. The chi-square (*Χ*^2^) p-value, degree of freedom (df), and number of observations (n) are shown in the lower left.

**Figure 4:**
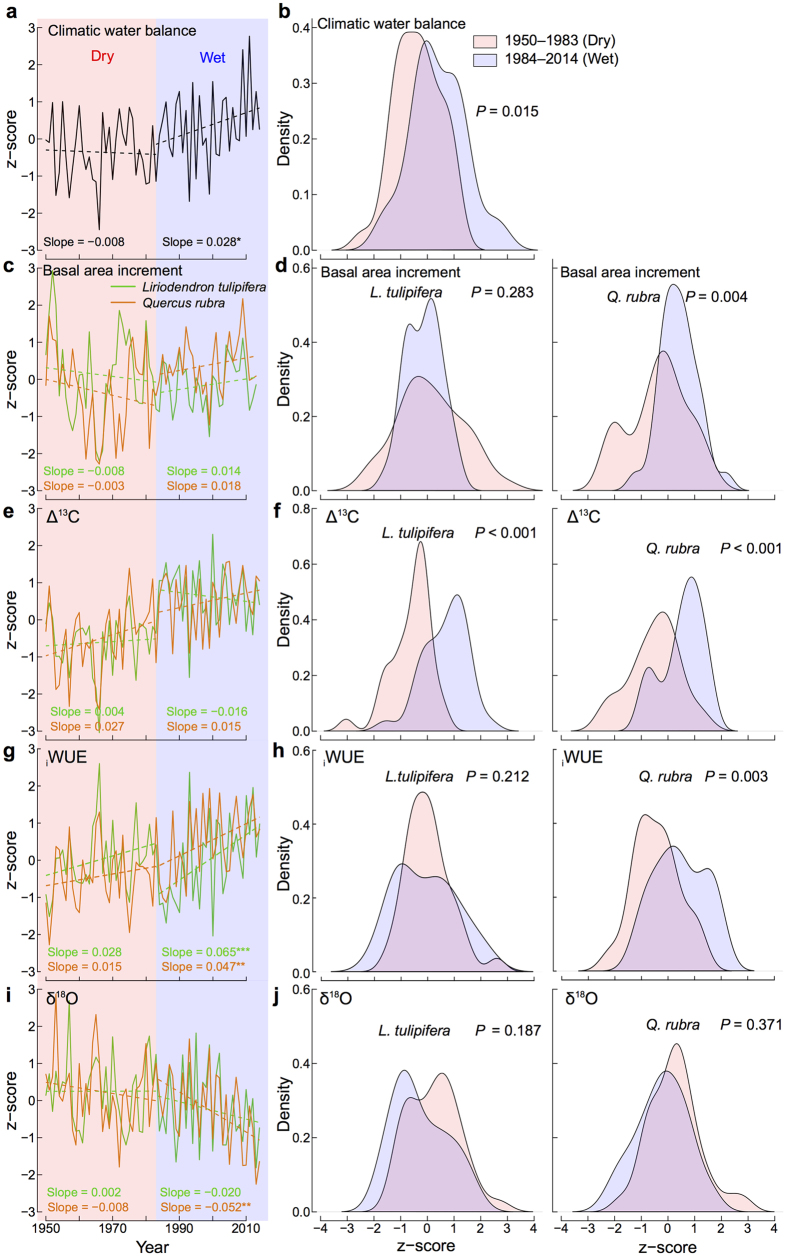
Trends and probability density functions. Climatic water balance (**a,b**), basal area increment (**c,d**), Δ^13^C (**e,f**), _i_WUE (**g,h**), and δ^18^O (**i,j**) of *Liriodendron tulipifera* and *Quercus rubra* for the dry 1950–1983 (light orange fill) and wet 1984–2014 (light blue fill) period. The dry and wet period were identified in the climatic water balance time series applying change-point detection test. Significance levels of the slopes from Mann-Kendall trend tests and Theil-Sen trend estimates: *P < 0.05; **P < 0.01; ***P < 0.001. *P*-values from the Kolmogorov–Smirnov tests between the dry and wet period are shown. Variables were standardized (z-score) before.

We performed piecewise structural equation modeling (SEM) using the R package *piecewise SEM*^54^ to account for concomitant changes in atm. CO_2_ concentration, SO_4_ and N deposition, and climatic water balance, which may be masking the influence of a single environmental variable on the growth and gas exchange of trees (Fig. 2). Piecewise SEMs use advanced multivariate statistical techniques better suited for small sample sizes and allow the simultaneous implementation of non-normal distributions, random effects, and different correlation structures within a traditional SEM framework^54^,^55^,^56^. We developed piecewise SEMs for each species to address the joint effects of changing climatic water balance, atm. CO_2_ concentration, and SO_4_ and N deposition on BAI, Δ^13^C and δ^18^O of trees. We considered the random tree identity effect (individual tree series) by fitting each response variable to a linear mixed effects model^57^ using the function lme from the *NLME* package^58^. The SEMs were fit for the period 1981–2014, i.e. the time window with available deposition data, and raw tree-ring and environmental data to account for effects of the trends in environmental variables. We assessed the models fits using chi-square p-value and Akaike’s information criterion corrected for small sample size^54^.

We used the change-point detection test of Pettitt^59^ to test the shift in the central tendency of the climatic water balance time series. Based on this test, we identified two significant periods with contrasting climatic water balance, i.e., the dry period 1950–1983 and the wet period 1984–2014. We assessed the significance of the changes in BAI, Δ^13^C, _i_WUE and δ^18^O in the dry vs. wet period with probability density functions and Kolmogorov–Smirnov tests. We estimated the temporal trends of the climatic water balance, BAI, Δ^13^C, _i_WUE and δ^18^O time series, and their significance with Mann-Kendall trend tests and Theil-Sen trend estimates^60^. All data analyses were conducted in R version 3.2.2^61^.

## Additional Information

**How to cite this article**: Levesque, M. *et al*. Water availability drives gas exchange and growth of trees in northeastern US, not elevated CO_2_ and reduced acid deposition. *Sci. Rep.* **7**, 46158; doi: 10.1038/srep46158 (2017).

**Publisher's note:** Springer Nature remains neutral with regard to jurisdictional claims in published maps and institutional affiliations.

## Supplementary Material

Supporting Information

## Figures and Tables

**Table 1:** Pearson’s correlation coefficients between tree-ring time series and June–August climate variables for the period 1950–2014.

Species	Tree-ring variable	Climatic variable			
		Maximum temperature	Precipitation	VPD_max_	Climatic water balance
*Liriodendron tulipifera*	BAI	−0.25*	0.37**	−0.40**	0.37**
	Δ^13^C	−0.59***	0.67***	−0.70***	0.73***
	_i_WUE	0.56***	−0.53***	0.67***	−0.57***
	δ^18^O	0.57***	−0.66***	0.71***	−0.71***
*Quercus rubra*	BAI	−0.44**	0.32*	−0.51***	0.36**
	Δ^13^C	−0.63***	0.56***	−0.70***	0.65***
	_i_WUE	0.52***	−0.31*	0.57***	−0.35**
	δ^18^O	0.41***	−0.48***	0.56***	−0.55***

Tree-ring and climate time series were standardized and prewhitened (removal of the first order autocorrelations) before correlation analysis.

VPD_max_, maximum vapor pressure deficit.

BAI, basal area increment.

_i_WUE, intrinsic water-use efficiency.

Significance levels: *P < 0.05; **P < 0.01; ***P < 0.001.
