# Supplementary material for: Water availability drives gas exchange and growth of trees in northeastern US, not elevated CO2 and reduced acid deposition
Source: Sci Rep. 2017 Apr 10;7:46158. doi: 10.1038/srep46158 (PMC5385545; doi:10.1038/srep46158)
Supplement: Supporting Information [file srep46158-s1.pdf]

**Supplementary Information**

**Article title:** Water availability drives gas exchange and growth of trees in northeastern US, not elevated $CO_2$ and reduced acid deposition

Authors: Mathieu Levesque, Laia Andreu-Hayles, Neil Pederson

The following Supplementary Information is available for this article:

**Table S1.** Tree characteristics and expressed population signals.
**Methods S1.** Calculations of $\Delta^{13}C$ and $_{i}WUE$.
**Methods S2.** $\delta^{18}O$ in precipitation data.
**Figure S1.** Air mass trajectories and $\delta^{18}O$ of precipitation.
**Figure S2.** Temperature and precipitation trends in the eastern US
**Figure S3.** Raw and residual basal area increments.
**Figure S4.** Correlations coefficients between *Liriodendron tulipifera* tree-ring time series and climate variables.
**Figure S5.** Correlations coefficients between *Quercus rubra* tree-ring time series and climate variables.

**Table S1** Number of trees sampled for tree growth and stable isotopes analysis, mean diameter at breast height (DBH), mean age, and expressed population signal (EPS[1]) of the mean tree-ring width (TRW) and isotope chronologies

| Species | No. of trees (growth) | No. of trees (isotopes) | DBH (cm) ±SD | Age ±SD | EPS | | |
|---|---|---|---|---|---|---|---|
| | | | | | TRW | $\Delta^{13}C$ | $\delta^{18}O$ |
| *Liriodendron tulipifera* | 15 | 5 | 67.6 ±11.5 | 126 ±9 | 0.93 | 0.92 | 0.85 |
| *Quercus rubra* | 15 | 5 | 49.4 ±12.6 | 138 ±14 | 0.92 | 0.86 | 0.89 |

[1]EPS provides an estimate of how closely a given mean tree-ring chronology based on a finite number of trees expresses its hypothetically perfect chronology based on an infinite number of trees. Chronologies with EPS ≥0.85 are considered adequate to reflect a common signal [1]. The EPS was calculated for the period of analysis 1950–2014.

**Methods S1**

**Calculations of Δ$^{13}$C and $_{i}$WUE**

Carbon isotope discrimination (Δ$^{13}$C) corresponds to the preferential use of lighter $^{12}$C atoms over the $^{13}$C heavier atoms during photosynthesis and it is calculated as the difference between the δ$^{13}$C of the air (δ$^{13}C_{air}$) and δ$^{13}$C in tree ring (δ$^{13}C_{tree}$). We used the linear interpolation technique from Leuenberger[2] to estimate values of δ$^{13}C_{air}$.

$$\Delta^{13}\mathrm{C}\ (‰) = \frac{(\delta^{13}\mathrm{C_{air}} - \delta^{13}\mathrm{C_{tree}})}{(1 + \frac{\delta^{13}\mathrm{C_{tree}}}{1000})} \qquad (1)$$

Δ$^{13}$C is linearly related to the ratio of intercellular $c_i$ to atmospheric ($c_a$) $CO_2$ mole fractions[3] via:

$$\Delta^{13}\mathrm{C}\ (‰) \cong a + (b - a)c_i/c_a \qquad (2)$$

where $a$ (4.4‰) is the fractionation during $CO_2$ diffusion through the stomata[4], and $b$ (27‰) is the fractionation during carboxylation in $C_3$ plants[5]. We used $c_a$ values based on ice core data for the period 1950–1957 and a yearly average of direct observations after 1957 (http://scrippsco2.ucsd.edu)[6] . $_{i}$WUE was estimated from values of Δ$^{13}$C and $c_a$ according to Farquhar & Richards[5]:

$$_{i}WUE = \frac{A}{g_s} = \frac{c_a(b - \Delta^{13}C)}{1.6(b-a)} \qquad (3)$$

where 1.6 is the ratio of diffusivities between water vapor and $CO_2$ in air.

Previous studies have reported that trends in Δ$^{13}$C and iWUE could potentially be related to increased tree height over time as Δ$^{13}$C can decline with increasing height[7] as a result of: adjustments in hydraulic conductivity[8]; assimilation of δ$^{13}$C-depleted air near the forest floor[9]; and changes in irradiance and photosynthetic capacity with height in the canopy[10]. To avoid this potential bias, we sampled mature trees (>125 yrs old, Table S1). Those trees were at least 65 year old at the beginning of the investigation period in 1950 and according to site index curves tree height of *Q. rubra* and *L. tulipifera* has only increased by 2-6 m. Such increase in tree height had no or only minor effects on tree-ring Δ$^{13}$C values[7]. Additional evidence is provided by studies showing that before the rise in atmospheric $CO_2$ concentration, trees generally do not show any age-related δ$^{13}$C trends after an initial juvenile phase of circa 50 years[11].

**Methods S2**

## $\delta^{18}O$ of precipitation

Variation in tree-ring $\delta^{18}O$ is primarily due to evaporative enrichment at the leaf level, biochemical fractionation during oxygen incorporation, and isotopic signature of tree source water, which is mainly influenced by the $\delta^{18}O$ of precipitation, and to a lesser extent from soil evaporative enrichment[12,13]. Therefore, when isolating the leaf level effect on oxygen isotope enrichment in tree rings a good understanding of $\delta^{18}O$ of tree source water / precipitation is necessary. Unfortunately, such records do not exits at our study site, so we had to rely on $\delta^{18}O$ of precipitation data from another site in northeastern US. We used the newly developed and longest (1968–2010) continuous record of precipitation isotope from the Hubbard Brook Experimental Forest (43º56′N, 71º45′W)[14] to help interpreting our tree-ring $\delta^{18}O$ results. The Hubbard Brook Experimental Forest is located ca. 300 km northeast from Black Rock Forest (Fig. S1a). The analysis of the air mass back trajectories (1968–2010) indicates that at both sites the moisture sources show some similarities despite some monthly offsets in $\delta^{18}O$ of precipitation due to differences in air temperature, latitude, and precipitation type (rain / snow) (Fig. S1b). Based on these exploratory analyses, we considered that the mean yearly $\delta^{18}O$ of precipitation records from Hubbard Brook Experimental Forest reflect those at Black Rock Forest. For the period 1968–2010, a significant reduction in $\delta^{18}O$ of precipitation (− 0.089‰ $yr^{-1}$) was recorded in northeastern US (Fig. S1c)[14] and was attributed mainly to the increases in the proportion of Arctic precipitation sources which are more depleted in $\delta^{18}O$[14].

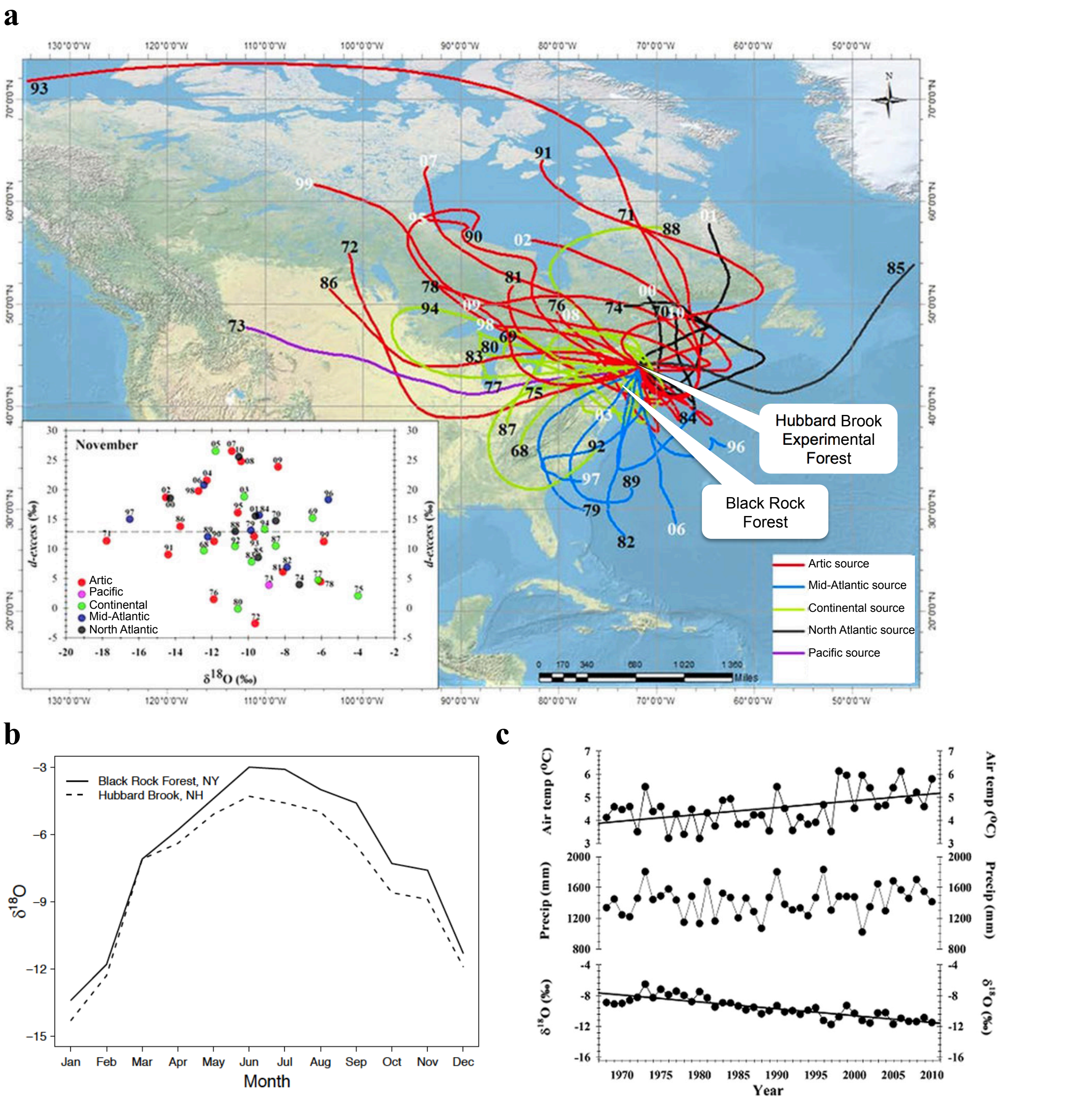


**Figure S1.** Air mass back trajectories and δ$^{18}$O- *d*-excess values (inset) for November for the period 1968–2010 in northeastern US **(a)**. Estimated mean monthly δ$^{18}$O of precipitation at Black Rock Forest and Hubbard Brook Experimental Forest **(b)**. Monthly estimated values were obtained from the Online Isotopes in Precipitation Calculator[15,16]. Trends in mean annual temperature, precipitation sum and δ$^{18}$O of precipitation at Hubbard Brook Experimental Forest **(c).** Results indicate a significant increasing trend in air temperature (slope = 0.03 ºC $yr^{-1}$, $P$ = 0.0017); no significant trend in precipitation amount (slope = 2.71 mm $yr^{-1}$, $P$ = 0.25); and significant declines in δ$^{18}$O of precipitation (slope = − 0.089‰ $yr^{-1}$, $P < 0.0001$). Figure panels **(a)** and **(c)** are adapted from Puntsag et al.[14] and are licensed under a Creative Commons Attribution 4.0 International License. The license terms can be found on the following link: http://creativecommons.org/licenses/by/4.0/.

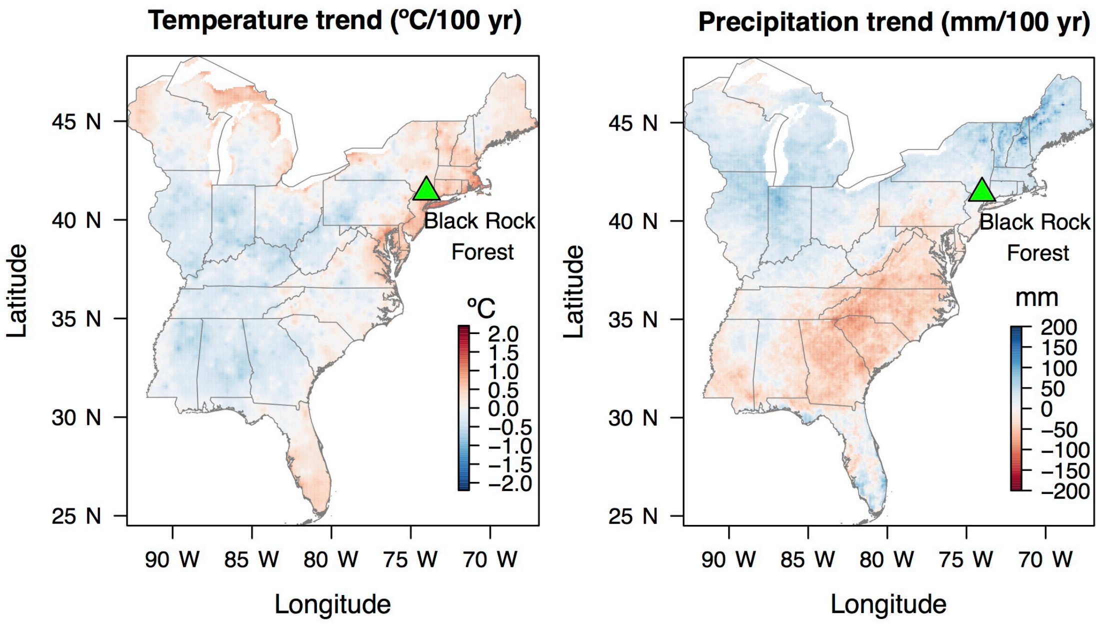


**Figure S2.** Observed temperature and precipitation trends in summer (June–August) for 1895–2015 in Eastern US. Figure created with R version 3.2.2[17].

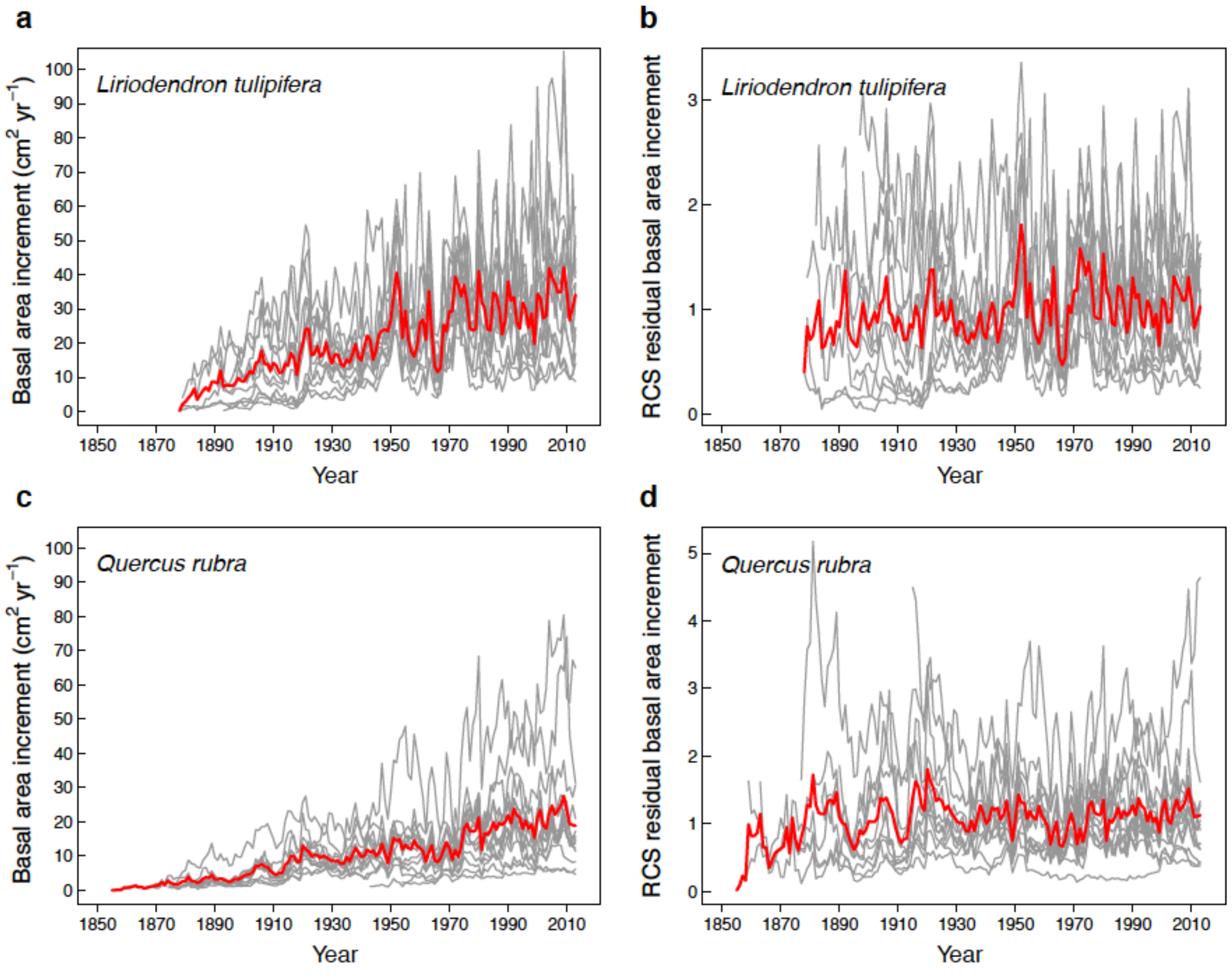


**Figure S3.** Raw basal area increment (BAI) series with the mean in red (**a,c**). Residual basal area increment series after Regional Curve Standardization (RCS) with the mean in red (**b,d**). In RCS, an average ontogenetic growth curve for *L. tulipifera* and *Q. rubra* (i.e., the regional curve) was calculated by aligning the raw BAI measurements of each tree to the biological age of the rings, and individual BAI series were then divided by this average curve[18,19].

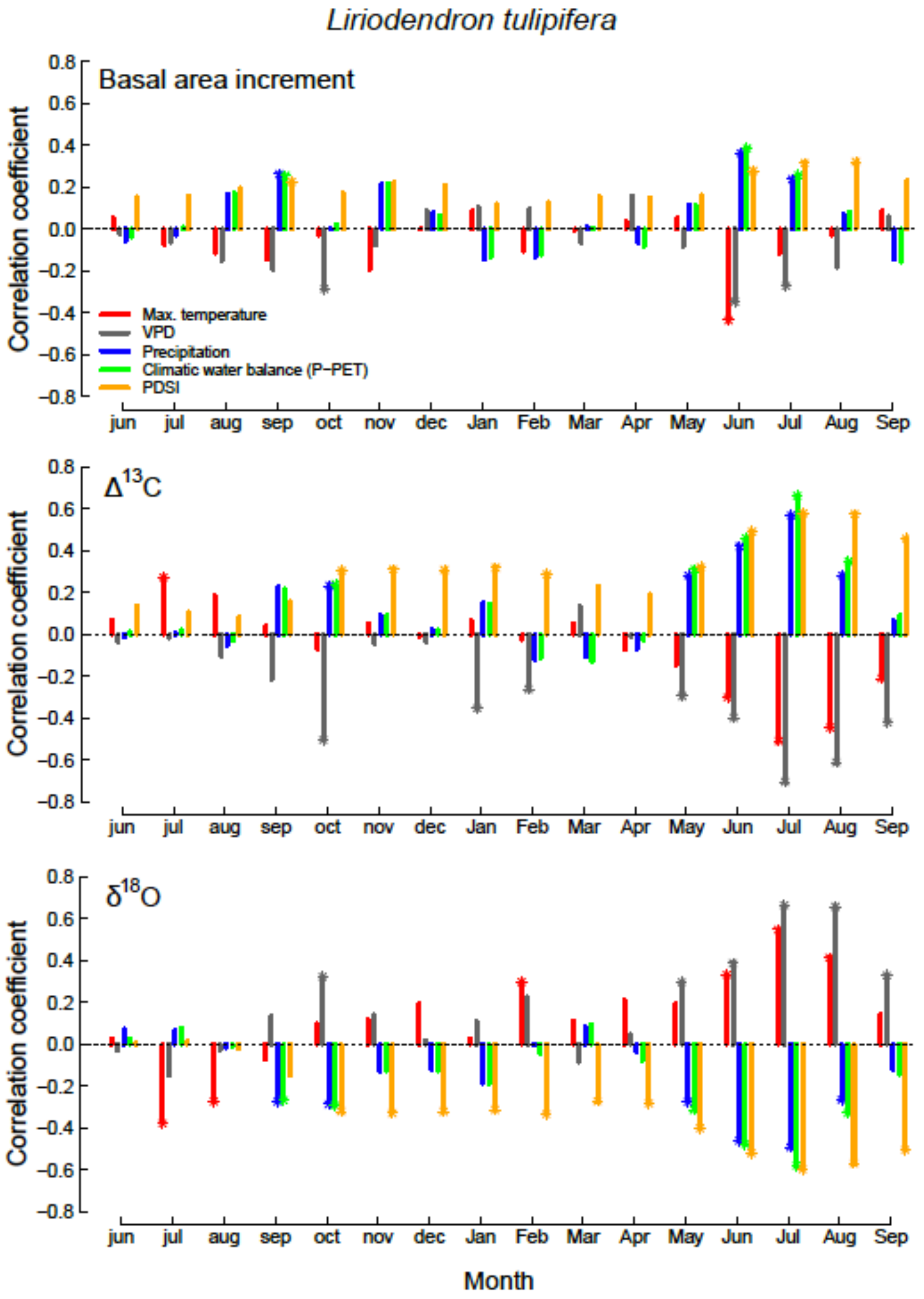


**Figure S4.** Bootstrapped Pearson's correlations coefficients calculated between *Liriodendron tulipifera* tree-ring time series (basal area increment, $\Delta^{13}C$ and $\delta^{18}O$) and monthly mean maximum temperature, vapor pressure deficit (VPD), precipitation sum, climatic water balance, and self-calibrated Palmer drought severity index (PDSI) time series for the period 1950–2014. Stars denote significant correlations ($P<0.05$). Months that are not capitalized are from previous year.

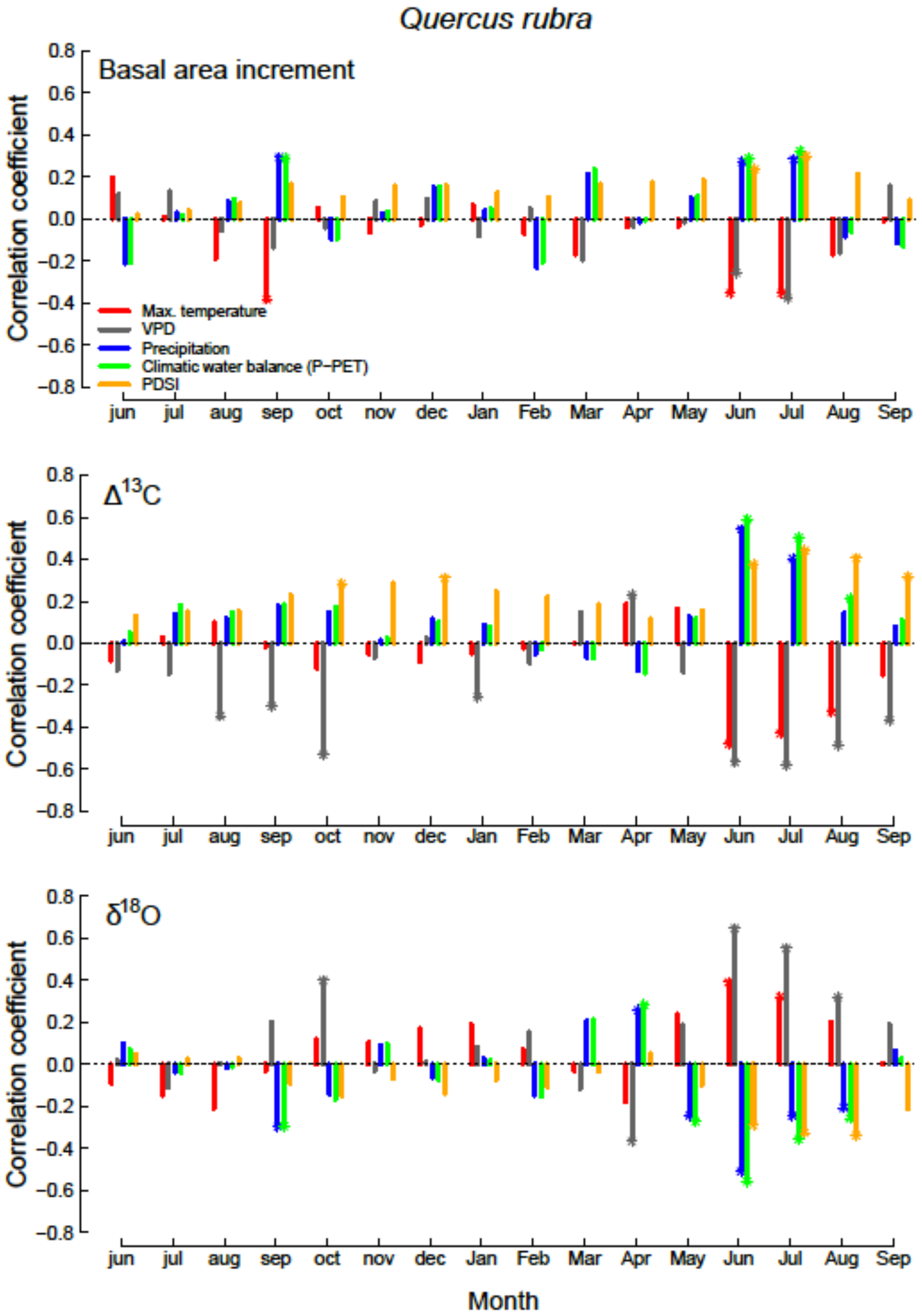


**Figure S5.** Bootstrapped Pearson's correlations coefficients calculated between *Quercus rubra* tree-ring time series (basal area increment, $\Delta^{13}C$ and $\delta^{18}O$) and monthly mean maximum temperature, vapor pressure deficit (VPD), precipitation sum, climatic water balance, and self-calibrated Palmer drought severity index (PDSI) time series for the period 1950–2014. Stars denote significant correlations ($P$<0.05). Months that are not capitalized are from previous year.

## References


1 Wigley, T. M. L., Briffa, K. R. & Jones, P. D. On the average value of correlated time series, with applications in dendroclimatology and hydrometeorology. *Journal of Climate and Applied Meteorology* **23**, 201–213 (1984).

2 Leuenberger, M. in *Stable Isotopes as Indicators of Ecological Change* Vol. Volume 1 (eds T. E. Dawson & R. Siegwolf) 211-233 (Academic Press, 2007).

3 Farquhar, G. D., O'leary, M. & Berry, J. On the relationship between carbon isotope discrimination and the intercellular carbon dioxide concentration in leaves. *Aust. J. Plant Physiol.* **9**, 121–137 (1982).

4 O'Leary, M. H. Carbon isotope fractionation in plants. *Phytochemistry* **20**, 553–567 (1981).

5 Farquhar, G. & Richards, R. Isotopic composition of plant carbon correlates with water-use efficiency of wheat genotypes. *Aust. J. Plant Physiol.* **11**, 539–552 (1984).

6 Keeling, R. F., Piper, S. C., Bollenbacher, A. F. & Walker, J. S. *Atmospheric $CO_2$ records from sites in the SIO air sampling network.* Carbon Dioxide Information Analysis Center, Oak Ridge National Laboratory, U.S. Department of Energy, Oak Ridge, TN, U.S.A (2009).

7 McDowell, N. G., Bond, B. J., Dickman, L. T., Ryan, M. G. & Whitehead, D. in *Size-and Age-Related Changes in Tree Structure and Function* (eds F. C. Meinzer, Barbara Lachenbruch, & T. E. Dawson) 255–286 (Springer, 2011).

8 Monserud, R. A. & Marshall, J. D. Time-series analysis of $\delta^{13}C$ from tree rings. I. Time trends and autocorrelation. *Tree Physiol* **21**, 1087–1102 (2001).

9 Schleser, G. & Jayasekera, R. $\delta^{13}C$-variations of leaves in forests as an indication of reassimilated $CO_2$ from the soil. *Oecologia* **65**, 536–542 (1985).

10 Francey, R. J. & Farquhar, G. D. An explanation of $^{13}C/^{12}C$ variations in tree rings. *Nature* **297**, 28–31 (1982).

11 Gagen, M. *et al.* Exorcising the `segment length curse': summer temperature reconstruction since AD 1640 using non-detrended stable carbon isotope ratios from pine trees in northern Finland. *The Holocene* **17**, 435–446, doi:10.1177/0959683607077012 (2007).

12 Barbour, M. M. Stable oxygen isotope composition of plant tissue: a review. *Functional Plant Biology* **34**, 83–94, doi:10.1071/fp06228 (2007).

13 Roden, J. S., Lin, G. & Ehleringer, J. R. A mechanistic model for interpretation of hydrogen and oxygen isotope ratios in tree-ring cellulose. *Geochimica et Cosmochimica Acta* **64**, 21–35 (2000).

14 Puntsag, T. *et al.* Arctic Vortex changes alter the sources and isotopic values of precipitation in northeastern US. *Scientific Reports* **6**, 22647, doi:10.1038/srep22647 (2016).

15 Bowen, G. The Online Isotopes in Precipitation Calculator, version 2.2. Available online (http://www.waterisotopes.org). (2013).

16 Bowen, G., Wassenaar, L. & Hobson, K. Global application of stable hydrogen and oxygen isotopes to wildlife forensics. *Oecologia* **143**, 337–348, doi:10.1007/s00442-004-1813-y (2005).

17 R Core Team. R: A language and environment for statistical computing. R Foundation for Statistical Computing, Vienna, Austria. URL https://www.R-project.org/. (2015).

18 Cook, E. R. & Briffa, K. R. in *Methods of Dendrochronology. Applications in the Environmental Sciences* (eds E.R. Cook & L.A. Kairiukstis) 97-162 (Kluwer Academic Publishers, 1990).

19 Briffa, K. R. *et al.* Fennoscandian summers from ad 500: temperature changes on short and long timescales. *Clim. Dynam.* **7**, 111-119, doi:10.1007/bf00211153 (1992).
